# ACL Reconstruction in Skeletally Immature Athletes: Current Concepts

**DOI:** 10.3390/medicina61040562

**Published:** 2025-03-22

**Authors:** Manish Attri, Riccardo D’Ambrosi, Luca Farinelli, Shahbaz S. Malik, Darren De Sa, Sachin Tapasvi, Christian Fink, Amit Meena

**Affiliations:** 1 Department of Orthopaedics, Santosh Medical College and Hospital, Ghaziabad 201009, India; 2 IRCCS Istituto Ortopedico Galeazzi, Via Galeazzi 4, 20161 Milan, Italy; 3 Department of Orthopedics, Marche Polytechnic University, 60121 Ancona, Italy; 4 Worcestershire Acute Hospitals NHS Trust, Charles Hastings Way, Worcester WR5 1DD, UK; 5 Division of Orthopaedic Surgery, Department of Surgery, McMaster University Medical Centre, Hamilton, ON L8N 3Z5, Canada; 6 The Orthopaedic Speciality Clinic, Pune 411001, India; 7 Gelenkpunkt—Sports and Joint Surgery, FIFA Medical Center of Excellence, 6020 Innsbruck, Austria; 8 Department of Orthopedics, Shalby Hospital, Jaipur 302021, India

**Keywords:** pediatric ACL, skeletally immature knee, ACL reconstruction, current concepts

## Abstract

ACL injury in skeletally immature patients remains a debatable topic in terms of its management, surgical choices and rehabilitation. The treatment preferences vary across the globe. Children are not little adults in terms of their physiology and anatomy. Hence, contemporary treatment inferred from the adult population does not give the same outcomes in pediatric patients. An in-depth study of specific challenges and difficulties is warranted to optimize the treatment strategies to cater to this group of patients. There is a paucity of literature giving long-term follow-up of ACLR in skeletally immature patients and no standardized guidelines are present for managing this group of patients. The authors have tried to summarize the current concepts for managing ACL injuries in skeletally immature patients through this article. Multiple lacunae and controversies exist in the knowledge regarding the optimum treatment of pediatric patients with ACL injuries who are comparatively more prone to ACL tears than their adult counterparts. Identifying the best mode of management of ACL tears in these skeletally immature patients is necessary. Level of evidence: Level IV.

## 1. Introduction

In recent years, the incidence of sports injuries has increased dramatically in pediatric and adolescent athletes, most likely due to increased sports participation in this age group, better recognition of injuries and high-intensity training throughout the year due to increased competition [1,2,3]. Skeletally immature patients require special consideration during the surgical reconstruction of ACL as the physis of the femur and tibia are open, still causing growth and development [4,5,6,7] of the bone, which can be damaged during surgery. The physeal damage might affect the growth of the limb, producing various linear and rotational deformities. A recent meta-analysis showed that this LLD can range from more than 10 mm in up to 2.1% of the operated cases and more than 20 mm in up to 0.5% of all the operated cases [8].

The surgery and rehabilitation protocols need to be differently crafted for these patients, taking into consideration the surgical method of ACL reconstruction used, patient demographics and skeletal age. Since younger patients are known to be more active than adults with ACL injury, the risk of premature cartilage degeneration, meniscal tear, graft rupture and revision surgery are considerably higher in children and adolescents as compared to adults [9,10,11]. There is a growing interest in surgical techniques, optimum graft choices, re-rupture rates, return to sports and growth disturbances after ACL reconstruction in this age group [12].

An in-depth study of specific challenges and difficulties is warranted to optimize the treatment strategies to cater to this group of patients. Through this article, the authors have tried to summarize the current concepts for managing ACL injuries in skeletally immature patients.

## 2. Risk Factors

Various risk factors can lead to ACL injuries. These are broadly categorized into intrinsic and extrinsic risk factors. The intrinsic risk factors comprise biomechanical, hormonal and anatomical risk factors.

The biomechanical factors include pivoting, deceleration and landing maneuvers, which are affected by posture, limb alignment and increased quadriceps activation. There is a significant association of ACL injury with a lower hamstring-to-quadriceps ratio and greater knee extensor muscle strength, which is physiologically present in adolescent females [13].

Anatomical risk factors include increased pelvic tilt, increased Q angle, increased femoral anteversion, decreased intercondylar notch width, increased posterior tibial slope and patella alta [14]. This anatomical configuration is also common in adolescent females, which may increase their risk of noncontact injuries.

Extrinsic factors include weather conditions, type of sport [15,16], type of footwear and interaction of footwear with playground surfaces. Hard playing surfaces, as seen in summer, increase the friction coefficient, which in turn increases the ACL strain. Playing on natural grass increases the risk of injury compared to heavyweight artificial turf. Other extrinsic risk factors include the amount of sports exposure, level of sport, sports season and equipment. The type of footwear, like cleats, may also increase the strain on the ACL [17].

## 3. Clinical Examination

A thorough history and physical examination are essential for diagnosing ACL injury. Lachhman, anterior drawer and pivot shift tests are required to assess these injuries. However, pain and swelling can affect the accuracy of these tests.

In young athletes with hemarthrosis, up to 65% have ACL rupture [18,19]. The pivot shift test is positive only in up to 35% of the patients when awake, but this positivity increases to 98% when the patients are under the effect of anesthesia [20]. These diagnostic tests should be performed on bilateral limbs, as children tend to have an increased joint laxity [19].

While examining these children, it is also essential to document the baseline deformities of the knee and limb-length discrepancy if present. MRI serves as a chief imaging tool for diagnosing ACL tears in children. It also aids in assessing concomitant knee injuries, usually seen in the menisci and cartilage of the affected knee in up to 50% of these patients [21].

Additionally, for the initial work, a 51-inch standing anterior posteriorly hip-to-ankle radiograph can also aid in the quantification of limb-length discrepancy and angular deformities if present in these children [22]; the skeletal age of these patients may be determined using a PA view of the left hand [23] or pelvic, elbow and calcaneal X-rays [24,25]. It is also important to record the Tanner staging and age at menarche to appropriately record the timing of peak growth velocity [26].

## 4. Management

### 4.1. Conservative and Delayed Operative Treatment

As children have a good healing potential and there is an associated risk of physeal damage with surgery, nonoperative treatment was the mainstay of managing children with ACL injuries historically.

However, conservative management is shown to be associated with increased rates of sports dropout (44–94%) because of persistent knee instability (20–100%). It is also associated with progressive meniscus (or 0.23 *p* = 0.006), cartilage damage and arthritic changes in the affected knee [27]. Early ACL reconstruction decreases the risk of secondary meniscal injuries and chondral damage in children and adolescents [28].

These changes are more common in children who are not compliant with post-injury activity modification and indulge in their sport even in the post-injury period. The re-surgery and graft failure risk is also higher in these patients [29].

A recent meta-analysis has shown that pathological joint laxity is decreased with early stabilization of the knee, and the return to sports is improved [30].

### 4.2. Treatment of Partial Tears

Partial ACL tears are commonly seen among children. They are frequently managed conservatively, but this treatment is successful only in patients where less than 50% of the ACL fibers are torn, and the pivot shift test is Grade A positive. For full, complete tears, operative treatment is regarded as the gold standard; however, there is very little evidence comparing surgical and non-surgical management for ACL injuries in children.

A recent study conducted on patients with ACL injuries showed satisfactory clinical results with rehabilitation alone. Amongst the participants, only half of the patients required surgical intervention in adulthood. It has been shown that the children managed by nonoperative treatment had a lower return to play, persistent instability and increased likelihood of meniscal tears. These findings were accurate even when the surgery was delayed [31].

Nonoperative treatment can be reserved for low-energy isolated ACL injuries in low-demand patients, particularly in the initial period or until skeletal maturity if instability persists [32,33].

The role of MRI in excluding associated injuries and patient-specific pediatric physiotherapy using a specific protocol is essential to making nonoperative treatment successful. Lifelong patient education, regular patient monitoring till skeletal maturity and ACL reconstruction surgery if the trial of nonoperative treatment fails are pivotal.

### 4.3. Complete Tears

To respect the growing physis and restore stability in young children with complete ACL tears, the surgeons have devised various instrumentation and reconstruction techniques. These techniques are broadly classified into physeal-sparing techniques, partial transphyseal and transphyseal techniques.

The physeal-sparing techniques may further be classified into extraphyseal and all-epiphyseal techniques.

In the literature, no technique has shown a clear-cut superiority for ACL reconstruction over the others. Evidence recommends using autograft for primary ACL reconstruction in patients with open physis [34]. These surgeries are not free of complications, and up to 9.6% of these patients need revision surgery, and 8% end up with ACL injuries of the contralateral knee [35].

Soft tissue allografts like hamstring tendons have been traditionally used, but the dimensions of these grafts are unpredictable, and small size in the pediatric population may limit successful reconstruction [36]. Much research is directed towards the quadriceps tendon autograft, which produces less donor site morbidity than patellar tendon bone and hamstring tendon autograft. However, the QT graft has its complications, such as the risk of delayed rectus tendon retraction and quadriceps weakness [37]. Gagliardi et al. showed excellent stability and favorable patient-reported outcomes after 2 years following all types of ACL reconstruction in adolescents using QT [38]. Traditionally, surgeons have estimated the skeleton age using methods such as SMR and have used it to select the treatment choice. Recently, a study proposed tables where the residual growth of each segment is estimated based on the bone age and gender of the patient. It is shown to be more reliable to guide the treatment when compared to Tanner staging to plan the type of surgery and avoid complications [39,40].

## 5. Graft Choice

Various factors, such as patients’ age, anatomical characteristics and participation in elite sports [41], influence the choice of grafts. Autografts are considered the gold standard for ACL reconstruction in the pediatric age group. The most commonly used grafts are the hamstring and the iliotibial band [42]. Other alternatives include quadricep tendon, bone-patella tendon-bone and allograft. Allograft use has declined, as there is a significantly higher graft failure rate in skeletally immature patients (OR 3.87) [43].

Hamstring graft is commonly used, as it has no risk of patella fracture or physeal damage and is easy to harvest. However, it has possible higher re-rupture rates, variations in graft size and inferior graft–host integration times compared to BTB [41,44].

BTB graft could be a good choice in Tanner stage 3 and 4 patients with a low risk of growth disturbances and graft failure and a good return-to-sports rate of 91.7 to 100% [45]. It has a rapid tissue integration time and uses ligaments instead of tendons for reconstruction [35,46]. It should be noted that BTB autografts have a maximum risk of arthrofibrosis compared to other autografts [47,48]. BTB graft is also associated with patella fracture, anterior knee pain and physeal injuries; hence, it is not recommended for patients with open growth plates.

Quadriceps tendon autograft has recently become a popular alternative for ACL reconstruction across all age groups [49,50] due to its predictable graft diameter >8 mm, which can easily be assessed on preoperative MRI [51]; lower surgical insult; lower risk of patella fracture; return to preinjury level of sports and favorable patient-reported outcome measures [52,53].

While HT could be a common choice for ACLR, BPTB and QT have significantly lower failure rates in adolescent athletes less than 18 years of age, with QT having the lowest failure rate among the three [54].

ITB autografts are most commonly used for ACL reconstruction in patients between 8 and 10 years of age [55]. They require little surgical insult to harvest; however, thigh asymmetry can be evident in these patients [56]. The role of internal bracing/graft augmentation techniques is yet to be assessed in children [57].

## 6. Surgical Techniques 

Table 1 shows various procedures for ACL reconstruction in skeletally immature athletes.

### 6.1. Physeal-Sparing Techniques

#### 6.1.1. Extraphyseal Iliotibial Band Allograft Reconstruction

In children with Tanner stages 1 and 2 with a skeletal age ≤ 11 years for girls and ≤12 years for boys, a (modified Macintosh) combined intra-articular and extra-articular iliotibial band reconstruction may be used [58]. This non-anatomical technique uses the central portion of the iliotibial band, which is harvested proximally and is left attached to Gerdy’s tubercle distally. The graft is taken posteriorly in an over-the-top position, passed below the intermeniscal ligament on the tibial side and sutured to the periosteum. The graft is sutured proximally to the periosteum overlying the femur and the intermuscular septum. The physes are completely spared, and this surgery is easy to revise. The reconstructed iliotibial band has an analogy with ALL that provides additional rotational stability [59,60].

A recent study reporting 23 years of follow-up showed that this technique had good functional outcomes, with a graft re-rupture rate of 6.6% at an average of 33.5 months postoperatively. A total of 48% of the patients noted thigh asymmetry and 1.6% of patients reported pain at the graft harvest side; none of the patients reported limb-length discrepancy or alignment issues in the postoperative period [61].

Extra physeal ACLR is a biomechanical superior option, as it restores symmetric and physiologic kinematic and kinetic function in a child with growing bones [62,63].

#### 6.1.2. All-Epiphyseal Technique

Anderson developed this technique, which was later modified by various other authors. In this hamstring, autografts and epiphyseal sockets are used with all-epiphyseal fixation. Grafts are pre-tensioned and undergo circumferential compression to minimize the amount of bone removal required [64].

Although Anderson [65] reported favorable outcomes in 12 patients with 4.1 years of mean follow-up, certain authors have reported various complications with graft rupture rates ranging from 4 to 17% [42,66], limb-length discrepancy [66] and angular deformities where few children had to undergo surgical correction for the same [67].

Although the technique has comparable outcomes to the other techniques, the risk of physeal damage is substantial; this risk of growth plate violation can be reduced by using three-dimensional intraoperative imaging [68].

### 6.2. Partial Transphyseal Reconstruction

This procedure is useful in children with Tanner stage 3; in this technique, an over-the-top position or all-epiphyseal tunnel is used for the femur and a vertical, central and transphyseal tunnel is used for the tibia. Few authors have shown that this technique avoids post-surgical limb-length discrepancy or angular deformities. Still, there is a high rate of developing cartilage and meniscal injuries in follow-up [69].

A recent case series that included 23 participants operated with all-epiphyseal femoral tunnel and trans physeal tunnel in a mean age group of 13 years reported overgrowth of the ipsilateral limb between 1 and 2 cm in 2 patients; 1 of them required surgical correction [70].

Another study, which included 24 participants, found that 16.7% of them developed growth disturbances, and amongst those who developed LLD, 66.5% had more than five years of growth remaining [71].

### 6.3. Transphyseal Reconstruction

This technique is useful in children with Tanner stage 3 and above. In this technique, physeal-respecting transphyseal reconstruction may be performed by removing the minimum amount of physeal tissue and using soft tissue grafts with metaphyseal fixation.

A recent study conducted on patients who had undergone transphyseal ACLR showed that 3% of these children underwent revision surgery at a mean of 3.6 years of follow-up. The mean IKDC and Lysholm knee scores were 90 and 91 points, respectively [72]. There was no recorded limb-length discrepancy or angular deformities, which has further been confirmed by various other authors [73,74].

A systemic review study compared extraphyseal/all-epiphyseal versus trans physeal techniques for ACL reconstruction and found that in 425 cases, Lysholm and Tegner activity scores were comparable between both the approaches; the authors also found that outcomes for non-surgical treatment were statistically poor. The authors reported no differences in terms of graft rupture, LLD, malalignment or symptomatic instability [75].

### 6.4. Lateral Extra-Articular Procedures (LEAPs)

Lateral extra-articular procedures comprise LET and ALLR. Along with ACLR, these techniques reduce graft failure rates and improve the rotational stability of the knee [76,77].

There are a few concerns that limit the generalizability of LEAPs with ACLR, including the possible risk of over-tightening and subsequent degeneration of the lateral compartment of the knee, lack of diagnostic tools to guide the decision for adding LEAPs and very little available literature on the anterolateral pediatric knee [78,79].

A recent study has shown the anterolateral pediatric knee to be inconsistent and underdeveloped, which needs further in-depth research [80].

LEAPs should be added to ACLR if two or more of the following are present: [74,75,81,82]

- Recurvatum > 10 degrees
- Hyper laxity
- Grade pivoting injury
- (Lateral compartment osteochondral trauma, meniscus tear, multi-ligament injury)
- Involvement in pivoting, collision or contact sports
- Children with poor neuromuscular control
- Children with limited rehabilitation capacity

Studies performed on pediatric cadavers have shown an improvement in rotatory instability, but clinical data on pediatric patients are lacking regarding the same [62].

Further research is needed into this aspect, and more is to be discovered regarding the increased recovery time, increased surgical morbidity, and increased risk of physeal injury when LEAPs are combined with ACLR in the pediatric and adolescent populations.

**Table 1:** Various procedures for ACL reconstruction in skeletally immature athletes.

Procedure	Description	Merits	Demerits
Physeal-sparing ACL reconstruction [83]	ACL graft is passed without crossing the growth plates, preserving their integrity This technique includes extra physical grafting and femoral tunnel placement away from the growth plate	It is done for children with terminal stages one and two as it minimizes the stress of growth plate disturbance Associated with Lori rupture rates, ensuring the durability of reconstruction during skeletal growth	Growth disturbances reported rates of 5.8%. Non-anatomical graft placement can compromise knee stability
All-epiphyseal over-the-top technique [84]	Graft passed over the back of the femoral condyle and the top position Extra-articular lateral tenodesis followed by graft fixation	It is ideal for skeletally immature athletes as a procedure that avoids growth plate damage Gold, clinical outcomes, improved diagnosis codes, and excellent knee stability	A high level of surgical skill is required High cost and complexity Main film line discrepancies up to 1 cm and mild axial deformities up to 4° of varus
All-inside ACL reconstruction [85]	Graft placement without creating large tunnels Graph secured using buttons or suture tensioning devices	Small incision It avoids large bone tunnels Preserves joint anatomy Quick recovery	Requires precise technique and specialized equipment It may not be appropriate for severe ACL injuries
Partial transphyseal reconstruction [83]	It uses a transphyseal tibial tunnel but avoids a femoral tunnel Used in borderline pubescent Tanner stage 3	Reduce the stress of lateral distal femur and physeal damage and angular deformities	A graft failure rate of 20 to 25%
Transphyseal construction [7,86]	ACL grafts pass through the growth plates of both the femur and tibia Used for Tanner stages 3 and 4	Allows anatomical graft placement Better rotational stability Good, long-term functional results	Risk of growth disturbances as graft placement crosses the growth plates It can cause steep graft angles as the patient grows

Surgical Considerations for Deformity Around the Affected Knee

Analyzing rotational deformities around the knee in pediatric patients with anterior cruciate ligament (ACL) tears preoperatively is crucial for effective surgical planning. These deformities can significantly impact knee biomechanics and influence both the risk of injury and the outcomes of ACL reconstruction.

The assessment of rotational deformities can be performed using clinical evaluation by gait analysis, which can reveal abnormalities such as in-toeing or out-toeing, which may indicate underlying rotational deformities [87] and assessment of alignment of the lower extremity, including hip rotation, femoral anteversion, and tibial torsion, provides insights into rotational alignment [87].

CT imaging is considered the gold standard for quantification of rotational deformities by allowing precise measurement of femoral anteversion and tibial torsion angles [88]. MRI, while primarily used to assess soft tissue structures, can also provide insights on bone morphology and alignment, contributing to the evaluation of rotational deformities [89].

Recognizing and addressing rotational deformities is essential during surgical planning. Failure to correct these abnormalities may result in persistent instability or graft failure post-reconstruction [90]. Proper identification and correction of rotational deformities are associated with improved functional outcomes and reduced risk of re-injury [91]. Preoperative imaging should also encompass assessments of the patellofemoral joint to identify any instability or malalignment that could influence surgical planning [92].

Addressing concurrent lower limb deformities in pediatric patients with anterior cruciate ligament (ACL) injuries requires meticulous preoperative assessment and strategic planning. The decision to correct these deformities in conjunction with ACL reconstruction hinges on their severity and impact on knee function.

This decision on when to address deformities relative to ACL reconstruction depends on their severity and functional implications. In cases of severe deformities having significant malalignments that adversely affect knee biomechanics, surgical correction either prior to or simultaneously with ACL reconstruction may be necessary to optimize outcomes and restore proper limb alignment and knee function, thereby enhancing the success of ACL reconstruction [93].

Mild to moderate deformities may be monitored and addressed during ACL reconstruction if they are deemed to impact knee stability or function. In such cases, a tailored approach is essential, considering the patient’s specific anatomical and functional characteristics to achieve optimal surgical outcomes [94].

A meticulous preoperative assessment and individualized treatment plan are vital for managing pediatric ACL injuries with concurrent lower limb deformities. The timing and approach to deformity correction should be carefully considered to ensure optimal knee function and long-term outcomes. The authors preferred a treatment algorithm for the ACL reconstruction technique based on Tanner staging and estimated remaining bone growth shown in Table 2.

## 7. Rehabilitation

The final outcome of ACLR is influenced by the child’s physiological and physical state, the surgical technique used, the magnitude of initial injury, the time since operation and accessibility to specialist facilities and professionals [95,96,97].

Amongst these, the most critical aspect for the success of ACLR is physiotherapy, but currently, for youth, specific rehabilitation protocols following ACL reconstruction surgery are scarce.

Return to preinjury sports amongst children is high, as are the injury rates amongst these patients [98]. Special consideration must be given to various features of growing athletes, such as the developing neuromuscular system, resulting in different pivoting and landing kinematics and kinetics, including a greater dynamic knee valgus force as compared to adults [99]; these unique factors make them different from their adult counterparts [100].

The existing rehabilitation protocols are designed with a combination of clinical expertise and adult literature [101,102,103].

Generally, these protocols involve the initial phase of controlling pain and edema and restoring knee ROM. This is followed by strengthening, non-impact activities, straight-line running, cardiovascular exercise, and a return to sports, which is allowed around 6 to 12 months after surgery. Focused hamstring strengthening exercises are included in the ongoing athletic training to decrease the risk of retear of the graft. An alternative is redirecting the athlete to lower-risk sports and activities [15]. Artificial intelligence and virtual reality can also aid in the rehabilitation process by providing closer guidance and rigid objective targets [104].

## 8. Prevention

ACL injury can have a significant impact on young athletes due to time out of sports and future and excessive cost of treatment. ACL injury prevention programs have gained popularity during the past decade and are of key interest to trainers, sports physicians and orthopedic surgeons. These programs comprise plyometrics techniques and strength and balance exercises with other neuromuscular training aimed at reducing the rate of ACL injuries. This program is aimed at training athletes for safer movement patterns that decrease the risk of injuries during high-risk sports by providing adequate control and avoiding dynamic valgus [31,105]. These programs decrease the risk of ACL injuries by 50% in all athletes, and their benefit is more apparent in female athletes, where this risk is reduced by two-thirds [106]. These programs are cost-effective and can be easily conducted with regular training 2 to 3 times a week. They also reduce the risk of re-ruptures in patients undergoing ACL reconstruction surgeries.

## 9. Conclusions

There has been an increased interest in ACL reconstruction in children and adolescents over the past few years. This has laid down the path for future research in this field. It is important to note that there has been a growing recognition of ACL injuries and various reconstruction techniques, emphasizing a return to sports, re-rupture rate, function and complications.

Thus, we conclude that “ACL re-rupture rates in this population are higher as compared to adults, so when these patients return to sports, a comprehensive evaluation of their sports ability, strengthening the monitoring of sports indicators and paying attention to sports attention is crucial”.

As the incidence of the pediatric population is on the rise, improvement in sports skills and sports-specific training, optimized playing conditions, and appropriate footwear is necessary, and early screening and diagnosis of ACL injuries should be strengthened.

The surgical techniques and outcomes of ACL reconstruction in adults cannot be mirrored in patients with open growth physis. Hence, research should be directed towards developing newer techniques and age-specific rehabilitation protocols in such patients.

## Figures and Tables

**Table 2:** The authors preferred a treatment algorithm for the ACL reconstruction technique based on Tanner staging and estimated remaining bone growth.

Tanner Stage	Estimated Bone Age	Remaining Linear Bone Growth	Recommend Technique
1, 2	<10 girls <12 boys	>7 cm >5 cm	All extra-articular technique All epiphyseal
3	<11–13 girls <13–15 boys	1–5 cm	Hybrid transphyseal
>=3	>14 girls >16 boys	<1 cm	Complete transphyseal

## Data Availability

No new data were created or analyzed in this study. Data sharing is not applicable to this article.
